# Longitudinal x-ray based lung function measurement for monitoring Nintedanib treatment response in a mouse model of lung fibrosis

**DOI:** 10.1038/s41598-023-45305-x

**Published:** 2023-10-30

**Authors:** Amara Khan, M. Andrea Markus, Angelika Svetlove, Swen Hülsmann, Frauke Alves, Christian Dullin

**Affiliations:** 1 Translational Molecular Imaging, Max Planck Institute for Multidisciplinary Sciences, Göttingen, Germany; 2 Cluster of Excellence “Multiscale Bioimaging: from Molecular Machines to Networks of Excitable Cells” (MBExC), Göttingen, Germany; 3 Department of Anesthesiology, University Medical Center, Göttingen, Germany; 4 Clinic of Hematology and Medical Oncology, University Medical Center, Göttingen, Germany; 5 Institute for Diagnostic and Interventional Radiology, University Medical Center, Göttingen, Germany; 6 Institute for Diagnostic and Interventional Radiology, University Hospital, Heidelberg, Germany; 7 Translational Lung Research Center, Heidelberg, Germany

**Keywords:** Preclinical research, Diagnostic markers

## Abstract

Lung fibrosis (LF) is a chronic progressive, incurable, and debilitating condition of the lung, which is associated with different lung disease. Treatment options are still sparse. Nintedanib, an oral tyrosine kinase inhibitor, significantly slows the LF progression. However, there is a strong need of further research and the development of novel therapies. In this study, we used a correlative set-up that combines X-ray based lung function (XLF) with microCT and whole body plethysmography (WBP) for a comprehensive functional and structural evaluation of lung fibrosis (LF) as well as for monitoring response to orally administered Nintedanib in the mouse model of bleomycin induced LF. The decline in lung function as early as one week after intratracheal bleomycin instillation was reliably detected by XLF, revealing the lowest decay rate in the LF mice compared to healthy ones. Simultaneously performed microCT and WBP measurements corroborated XLF findings by exhibiting reduced lung volume $V^{insp}_{\mu CT}$ and tidal volume $TV_{WBP}$. In LF mice XLF also revealed profound improvement in lung function one week after Nintedanib treatment. This positive response to Nintedanib therapy was further substantiated by microCT and WBP measurements which also showed significantly improved $V^{insp}_{\mu CT}$ and $TV_{WBP}$ in the Nintedanib treated mice. By comparing the XLF data to structural features assessing the extent of fibrosis obtained by ex-vivo high-resolution synchrotron radiation-based imaging and classical histology we demonstrate that: (1) a simple low dose x-ray measurement like XLF is sensitive enough to pick up treatment response, (2) Nintedanib treatment successfully improved lung function in a bleomycin induced LF mouse model and (3) differences between the fully restored lung function and the partially reduced fibrotic burden compared to healthy and untreated mice. The presented analysis pipeline underlines the importance of a combined functional and anatomical readout to reliably measure treatment response and could easily be adapted to other preclinical lung disease models.

## Introduction

Lung fibrosis (LF) is a chronic pathological condition of the lung, which can be caused by different diseases. It is characterized by the scarring of lung tissue and the accumulation of extracellular matrix (ECM) components that lead to pulmonary dysfunction and failure^1^. LF in patients is currently incurable, however treatment with (1) the tyrosine kinase inhibitor Nintedanib and (2) the pyridone derivative pirfenidone has shown promising outcomes for disease management and improving quality of life. Nintedanib slows fibrosis progression and reduces the decline in forced vital capacity (FVC) via its anti-fibrotic, anti-inflammatory and anti-angiogenic properties^2,3^. Interestingly, Nintedanib has also been successfully trialed in the therapy of Covid 19-induced lung fibrosis^4^.

Pre-clinical mouse models serve as vital tools to investigate the pathogenesis, reliable diagnostic techniques and therapies for LF, despite their limitation to mimic the irreversible nature of human LF^5^. Bleomycin induced LF is one of the most common animal models. Bleomycin is an antibiotic isolated from *Streptomyces verticillatus*, which is used as a chemotherapeutic agent to treat germinative tumors and lymphomas in humans. However, the use of bleomycin therapy is limited due to its pro-fibrotic properties, leading to the development of LF in 3–5 percent of patients receiving it^6,7^. This “side effect” has been exploited in rodents, where upon direct instillation in the lung it acts as a strong oxidative agent causing lung injury by DNA breakage and production of reactive oxygen species (ROS). The initial inflammatory phase is followed by a peak fibrotic response which is resolved over-time^8^.

In preclinical studies, lung function assessment during fibrosis progression is mainly performed using whole-body plethysmography (WBP) or micro-computed tomography (CT). The standardization of the parameters derived from unrestrained whole-body plethysmography (UWBP) is however, often debated^9–11^. Other techniques such as forced oscillation requires invasive intubation/tracheotomy which typically tends to be terminal^12^. Alternatively, microCT provides reliable quantitative evaluation of the extent of fibrosis and effect of therapeutic intervention but it requires a high radiation dose posing it unsuitable for longitudinal applications^13,14^. Since typically retrospective gating is employed, the lung gets reconstructed at only a few time points in the respiratory cycle, often at end-inspiration and end-expiration. Here we use the same approach of retrospectively gated CT for comparison. In contrast to that, we established X-ray-based lung function (XLF) that is derived from fast and low dose radiographic measurements and has shown high sensitivity in mouse models of chronic and inflammatory pulmonary diseases^15,16^. Furthermore, we show that regular planar XLF measurements can now also be performed with microCT in a single acquisition for direct evaluation of lung function, volume and anatomy^17^.

In order to evaluate the feasibility of the XLF technique for preclinical monitoring of the progression of fibrosis and the response to Nintedanib therapy, we used our novel correlative lung function measurement set up^18^ to perform XLF, microCT and WBP measurements in a mouse model of bleomycin induced lung fibrosis. The impaired lung function due to fibrosis progression, and also the positive response to Nintedanib treatment were reliably detected by XLF. Simultaneously performed microCT and WBP corroborated the XLF results. Furthermore, we found a strong correlation between in-vivo functional data and the extent of fibrosis evaluated ex-vivo by high-resolution synchrotron radiation based microCT imaging as well as by classical histology, which also showed a decrease in fibrosis following Nintedanib intervention. This demonstrates that XLF is a simple and reliable preclinical tool for longitudinal assessment of the course and severity of lung fibrosis as well as for the evaluation of therapeutic efficacy of anti-fibrotic therapies such as Nintedanib in mice.

## Results

### XLF reveals improved lung function in Nintedanib treated mice

**Figure 1:**
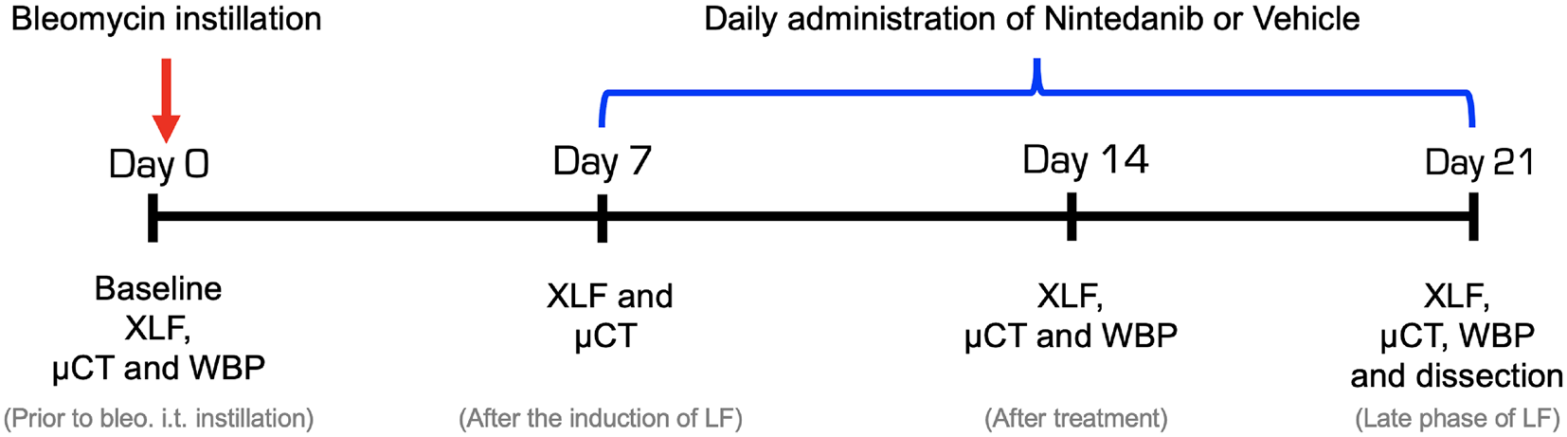
Experimental timeline for the preclinical mouse study. Bleomycin i.t. instillation (day 0) and oral vehicle (1% Tween 80-PBS) and Nintedanib treatment (daily from day 7 until day 20) as well as the time points of XLF, WBP and microCT measurements are shown in days. Bleomycin on day 0 was instilled i.t. immediately after performing the baseline scans and vehicle or Nintedanib on day 7 and 14 was administrated orally after the scans.

**Figure 2:**
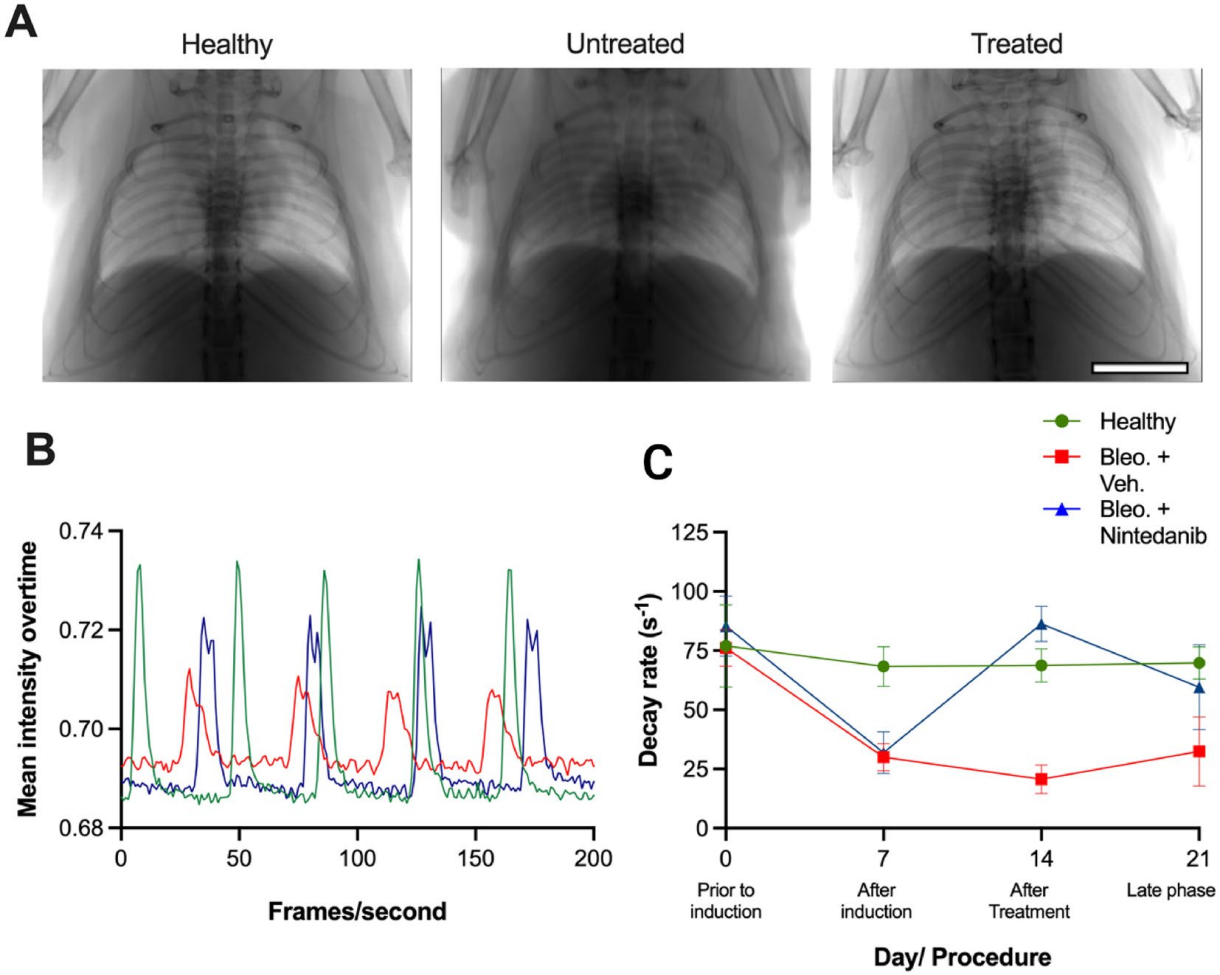
Radiographic XLF measurements performed in healthy, vehicle treated (bleo. + vehicle) and Nintedanib treated mice (bleo. + Nintedanib). (**A**) Radiographic Anterior Posterior projection of the chest region presenting the differences in x-ray attenuation at the lungs in all groups on day 21. (**B**) Graph showing representative x-ray transmission curves for four breathing cycles acquired at late phase of LF (day 21) for all groups. (**C**) Graph of decay rates for healthy (green), as well as bleo. + vehicle (red) and bleo. + Nintedanib treated LF mice (blue) at different time points. On day 0, a single dose of bleomycin was instilled i.t. following baseline lung function measurements and decay rates 7 days after LF induction, vehicle (1% Tween 80/PBS) or Nintedanib treatment, both orally administrated daily until day 21. Scale bar: 0.5 cm (**A**).

To assess the sensitivity of XLF for detecting fibrosis progression and response to Nintedanib treatment, XLF was performed on healthy mice as well as on mice with bleomycin induced lung fibrosis (LF) treated with either vehicle or Nintedanib following the experimental timeline shown in Fig. 1. The Nintedanib treatment was well tolerated by all mice. The overall differences in x-ray attenuation over the lungs in all three groups was evident in the anterior-posterior (AP) x-ray projections (Fig. 2A). This was successfully reflected in the x-ray transmission curves shown for four breathing cycles acquired at the late phase (day 21) which predominantly displayed an overall high x-ray attenuation at the lung of the vehicle (bleo. + vehicle) treated mice (Fig. 2B). Peak x-ray transmission representing the inhalation phase of the breathing cycle was lowest in the vehicle treated mice while the baseline in the expiratory phase was highest. This high x-ray attenuation baseline in the vehicle group indicates either a high consolidation of collagen, immune cell infiltration and/or partial collapse of airways. The Nintedanib treated LF group (bleo. + Nintedanib) showed a higher x-ray transmission at the peak of inspiration as compared to the vehicle treated group and a baseline similar to that of the healthy mice. Moreover, the total area under the curve (AUC) in the Nintedanib treated mice was higher than in vehicle treated ones (Fig. 2B). Compared to the healthy group, the Nintedanib treated group had a smaller AUC owing to a lower peak at inhalation which indicates an only partial improvement in the overall lung function (Fig. 2B).

XLF-derived decay rates in the expiration phases revealed the changes in the elastic recoil of the lung over three weeks in vehicle and Nintedanib treated mice. The baseline measurements for all groups showed relatively similar values for decay rate (Fig. 2C). One week after the instillation of bleomycin and before therapeutic intervention, mice belonging to the bleo. + vehicle and bleo. + Nintedanib group revealed a significant decline in decay rates (*p* = 0.02 and 0.03, respectively), whereas the healthy group had only negligible changes in decay rate over time. On day 14, following 7 days of therapeutic instillation, bleo. + Nintedanib treated mice exhibited a significant improvement in the decay rate as compared to the bleo. + vehicle treated LF group (*p* = 0.02) which points to a recovery from fibrosis in Nintedanib treated mice. In comparison, the bleo. + vehicle treated mice had a persistent low decay rate at day 14 which improved by the late phase (day 21). At day 21 the bleo. + vehicle group showed a mild improvement in the decay rate, which might indicate the start of the well-known recovery process in the bleomycin model. Collectively these results show that XLF allows monitoring of lung function related parameters over time. In particular, XLF demonstrates a significant lung function decline after bleomycin application and a clear improvement of lung function following 7 days of Nintedanib treatment.

### MicroCT and WBP measurements validate the results from XLF

**Figure 3:**
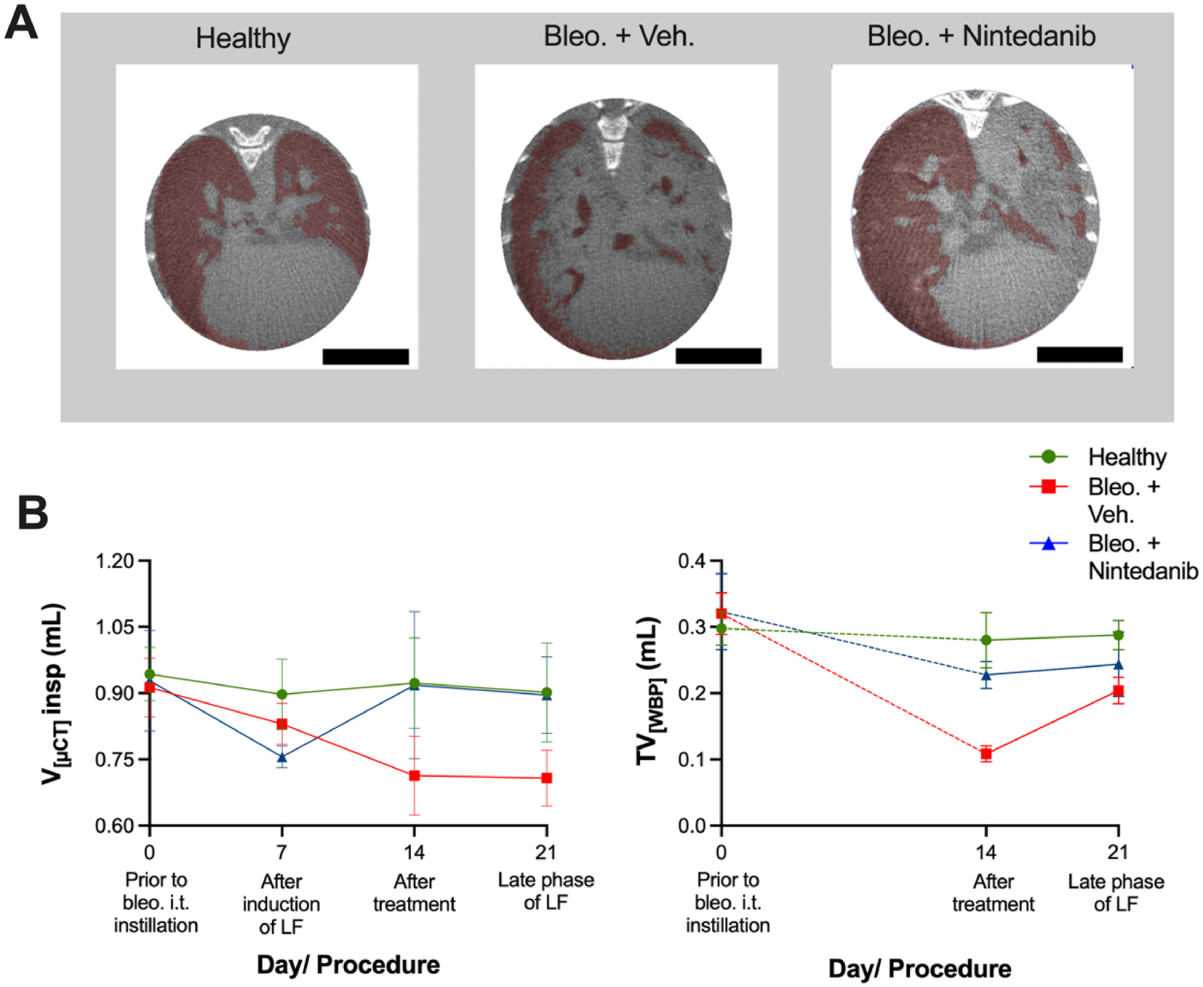
Lung volume assessment using microCT and WBP in healthy, vehicle treated LF (bleo. + vehicle) and Nintedanib treated LF mice (bleo. + Nintedanib). (**A**) The microCT measured lung volume at inspiration ($V^{insp}_{\mu CT}$) (red masked regions) derived from the segmented aerated regions of the lung through a region growing method is shown for a healthy, bleo. + vehicle and bleo. + Nintedanib treated LF mouse. (**B**) Graphs showing microCT measured $V^{insp}_{\mu CT}$ and WBP acquired tidal volume ($TV_{WBP}$) for healthy (green), bleo. + vehicle (red) and bleo. + Nintedanib treated LF mice (blue) at different time points. Scale bar: 1 cm (**A**).

A customized correlative set-up was used to perform microCT or WBP simultaneously with XLF measurements^18^. MicroCT data was reconstructed with a modified Feldkamp reconstruction algorithm to derive segregated lung volumes $V_{\mu CT}$ for each inspiration $V^{insp}_{\mu CT]}$ and expiration $V^{exp}_{\mu CT}$ phase. Using a region growing segmentation approach, the voxels corresponding to the air-filled regions of the lung were selected for determining $V^{insp}_{\mu CT}$ and expiration $V^{exp}_{\mu CT}$ respectively, while the dense tissue structures were not selected. The resulting segmentations revealed a low air content in all lung lobes of the bleo. + vehicle at late phase of LF (21 days) (mean $V^{insp}_{\mu CT}$ = 0.693 ± 0.074 ml) (Fig. 3A and B). The bleo. + Nintedanib group, as opposed to the vehicle group, possessed more air-filled regions (mean $V^{insp}_{\mu CT}$ = 0.896 ± 0.087 ml) similar to that of the healthy group (Fig. 3A and B).

The $V^{insp}_{\mu CT}$ showed a constant decline overtime following bleomycin installation in the bleo. + vehicle group (Fig. 3B). The $V^{insp}_{\mu CT}$ on day 21 for the bleo. + vehicle was significantly lower than both the healthy and the bleo. + Nintedanib treated group (*p* = 0.002 and 0.008, respectively) (Fig. 3B). The changes in the $V^{insp}_{\mu CT}$ overtime for the bleo. + Nintedanib group were comparable to changes of decay rate measured by XLF (Fig. 2C). As compared to the healthy group, the bleo. + Nintedanib group showed a decrease in $V^{insp}_{\mu CT}$ prior to the therapeutic intervention on day 7 (*p* = 0.11) which improved dramatically one week after the Nintedanib treatment (i.e. day 14) and matched the $V^{insp}_{\mu CT}$ values obtained for the healthy group (Fig. 3B). In the bleo. + vehicle treated group, WBP also exhibited a significantly lower tidal volume ($TV_{WBP}$) than the healthy and Nintedanib treated group on day 14 (*p* = 0.006 and 0.0110, respectively). In contrast to $V^{insp}_{\mu CT}$ measured by in-vivo microCT, which showed a less rapid decline between day 14 and day 21 but still smaller values at day 21 in the bleo. + vehicle, WBP showed an increase in $TV_{WBP}$ on day 21 (Fig. 3B). Interestingly, the increase in $TV_{WBP}$ of the bleo. + vehicle mice, is similar to that of the XLF measured decay rate, which also showed an increase in bleo. + vehicle treated mice by day 21 but to a significantly larger extent (Figs. 2C and 3B). Thus, it can be speculated that the assessment of lung function by WBP and XLF is more sensitive for detecting the early onset of the healing process in the untreated bleo. + vehicle group than in-vivo microCT, which only depicts anatomical changes.

### Ex-vivo synchrotron $\upmu$CT confirms a reduction in the amount of fibrotic tissue in response to Nintedanib treatment

**Figure 4:**
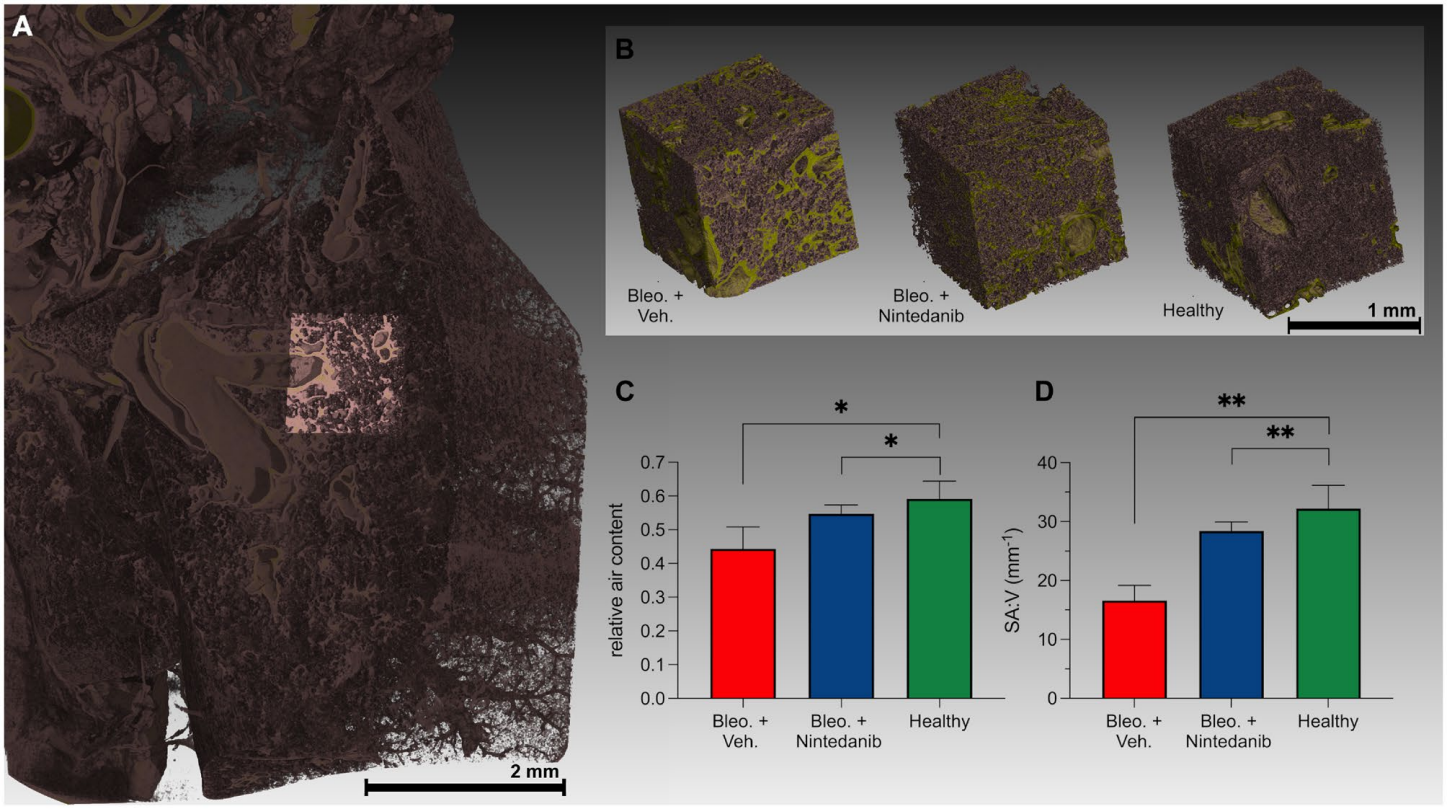
Ex-vivo SR$\upmu$CT imaging of explanted lungs obtained from healthy, bleo. + vehicle and bleo. + Nintedanib treated mice. (**A**) Reconstructed SR$\upmu$CT image showing ROI selection in one of the lung lobes. (**B**) 3D rendering of exemplary ROI’s of bleo. + vehicle, bleo. + Nintedanib and healthy mice (left to right). In green, dense tissue is displayed showing decreasing levels of fibrosis. (**C**) Graphs showing the relative air content in the measured cubic ROI’s, demonstrating a significantly reduced air content in bleo. + vehicle and bleo. + Nintedanib compared to the healthy control mice. (**D**) Graph showing the even more dominant differences in SA:V of the lung tissue to air interface. A *p* of 0.05 (*) was set as margin for statistical significance. (**) represents a *p* $< 0.01$.

Owing to the relatively poor resolution of the in-vivo microCT data, minute tissue structures cannot be resolved, hence, compromising the accuracy of the $V_{\mu CT}$. We therefore performed ex-vivo high-resolution synchrotron radiation based microCT imaging (SR$\upmu$CT) of whole lungs explanted from healthy (N = 3), bleo. + vehicle (N = 3) and bleo. + Nintedanib (N = 4) treated mice on day 21. From the reconstructed whole lung scans, an average of 6 ROIs with a size of 1.75 × 1.75 × 1.75 m$^{3}$ were selected in each lung specimen and surface determination was performed to delineate the boundaries of the lung tissue based on the grey scale values as indicated in Fig. 4A. Following a simple threshold based segmentation, relative air volume and lung surface area normalized to the lung tissue volume within the cube (SA:V) were calculated. The difference between cubes selected from the three different groups is depicted exemplary in Fig. 4B. Both bleo. + Nintedanib treated mice (mean air ratio = 0.547 ± 0.026) and healthy mice (mean air ratio = 0.592 ± 0.053 ml) displayed significantly higher air ratio than to the bleo. + vehicle group (mean air ratio = 0.444 ± 0.065 ml, *p* = 0.014 and 0.020, respectively) (Fig. 4C). Moreover, the SA:V of all the tissue components, was also significantly higher in the bleo. + Nintedanib treated (mean SA:V = 28.41 ± 0.1.55/mm) and healthy mice (mean SA:V = 32.21 ± 2.95/mm) than in the vehicle treated group (mean SA:V = 16.61 ± 2.56/mm, *p* = 0.0072 and 0.0029, respectively) (Fig. 4D).

**Figure 5:**
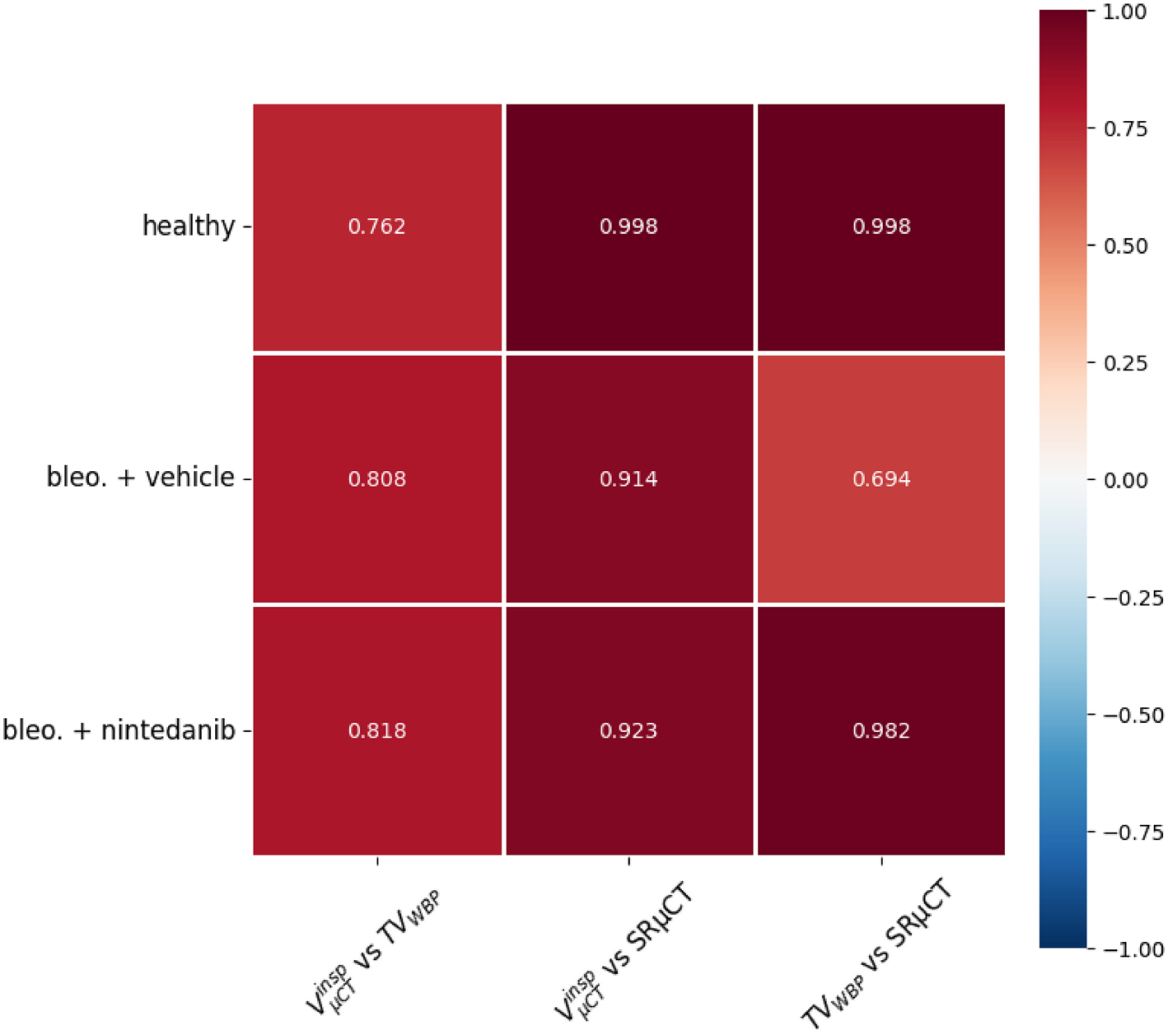
Heatmap showing spearman rank correlation coefficients between in-vivo and ex-vivo volumetric parameters. Spearman rank correlation coefficient (r) values are shown for in-vivo $V^{insp}_{\mu CT}$ vs in-vivo $TV_{WBP}$ in healthy, bleo. + vehicle and bleo. + Nintedanib treated mice for all days. Pearson correlation coefficient (r2) values are shown for in-vivo $V^{insp}_{\mu CT}$ vs ex-vivo SR$\upmu$CT and in-vivo $TV_{WBP}$ vs ex-vivo SR$\upmu$CT in healthy, bleo. + vehicle and bleo. + Nintedanib treated mice for day 21. Both WBP and in-vivo microCT data correlate well with each other and with the ex-vivo SR$\upmu$CT analysis.

In-vivo volumetric parameters including $V^{insp}_{\mu CT}$ and WBP were compared for all days to determine the reliability of data obtained as illustrated in the correlation heatmap in Fig. 5 (left column). $V^{insp}_{\mu CT}$ vs $TV_{WBP}$ displayed a strong correlation for all days in healthy (r = 0.762), untreated LF (r = 0.808) and Nintedanib treated LF mice (r = 0.818). Moreover, to assess the correlation between ex-vivo SR$\upmu$CT data and in-vivo functional data, we also compared SR$\upmu$CT tissue volume with $V^{insp}_{\mu CT}$ and $TV_{WBP}$ data obtained on day 21 (Fig. 5 middle and right column respectively). A strong correlation was found between SR$\upmu$CT tissue volume and $V^{insp}_{\mu CT}$ in healthy (r2 = 0.998), vehicle treated LF (r2 = 0.914) and Nintedanib treated LF (r2 = 0.923). $TV_{WBP}$ also showed strong correlation with SR$\upmu$CT in healthy (r2 = 0.998) and Nintedanib treated LF (r2 = 0.982) but only a moderate correlation in vehicle treated mice (r2 = 0.694). In summary, these correlations demonstrate that despite the limited spatial resolution the parameters extracted from in-vivo microCT are in good agreement with SR$\upmu$CT, which provides highly resolved structural details of the lung.

### Histological validation

**Figure 6:**
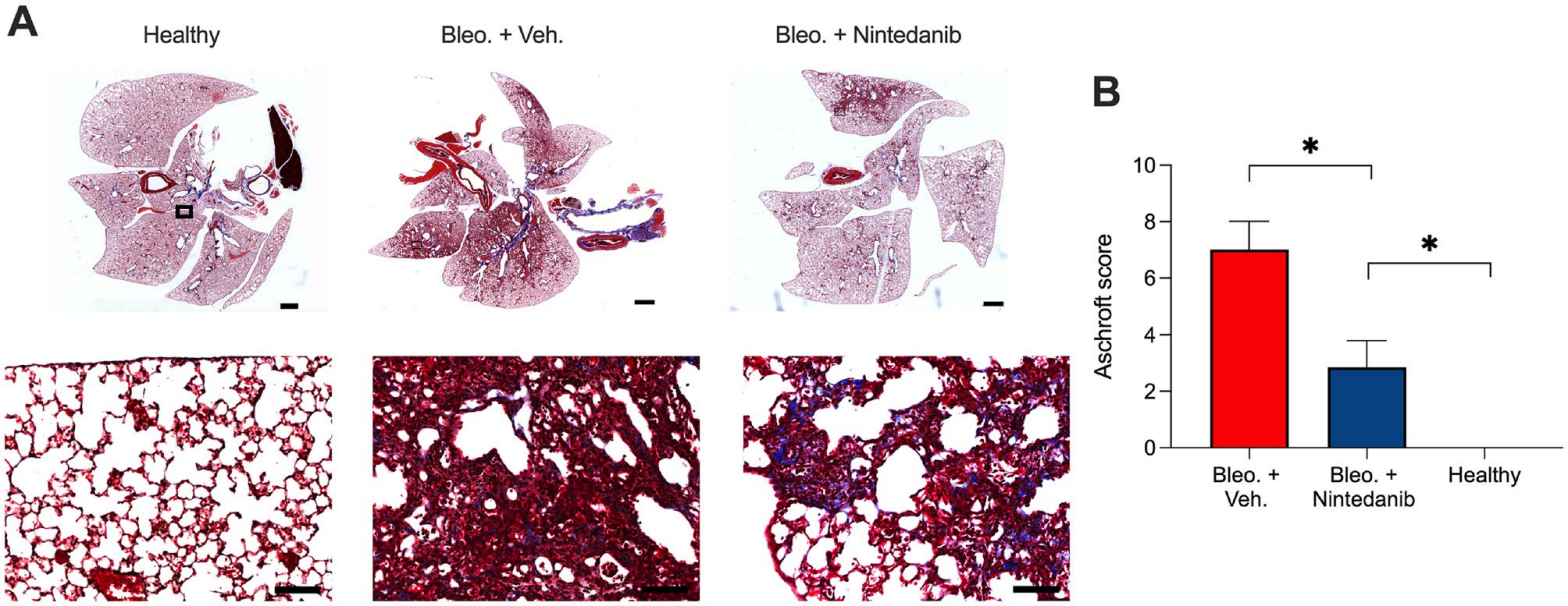
Histological assessment of lungs of healthy, bleo. + vehicle and bleo. + Nintedanib treated mice. (**A**) MTS stained representative overview images of whole lung paraffin sections (upper panel) and magnified images (20x, lower panel) showing the lung morphology at late phase (day 21) in lung tissue sections obtained from healthy mice, vehicle and Nintedanib treated LF mice. Magnified images are acquired at the ROI marked by black boxes (upper panel) and show the collagen stained in blue (lower panel). (**B**) Graph showing differences in the histological score for all three groups, demonstrating significantly higher scores for bleo. + vehicle group than healthy and Nintedanib treated group correlating to the extent of fibrosis. Note, that in the lungs of Nintedanib treated LF mice moderate fibrotic alterations of lung parenchyma are still present with a mean elevated score compared to healthy control. A *p* of 0.05 (*) was set as margin for statistical significance. (**) represents a *p* $<~0.01$, Scale bars: 1 mm for overview images (upper panel) and 200$\upmu$m for magnified images (lower panel).

Lastly, conventional histological analysis in combination with Masson trichrome staining (MTS) was performed to further validate the in-vivo and ex-vivo results in correlation to the extent of fibrosis (Fig. 6A). As expected, the healthy group exhibited a normal lung with minimal parenchymal changes. Bleomycin application resulted at day 21 in patchy fibrotic alterations of the lung parenchyma characterized by fibroproliferative foci with various degrees of confluence (Fig. 6A). MTS staining of lung sections of the bleo. + Nintedanib treated mice revealed substantially better restoration of the lung architecture than the bleo. + vehicle treated mice, however in comparison to healthy controls Nintedanib treated lungs retained residual fibrotic areas expressing collagen and extracellular matrix. Further examples can been found in supplemental material S2. The histological score among the five lobes, representing the extent of fibrosis, was performed on MTS stained lung tissue sections (Fig. 6B), which revealed significantly higher scores 7.01 ± 2.00 for the fibrotic masses in the vehicle treated LF group as compared to Nintedanib treated LF 2.85 ± 2.48 and the healthy group 0.00 ± 0.00 (p = 0.006 and p = 0.02 respectively). In summary, Nintedanib resulted only in a partial anti-fibrotic effect as the treated mice still exhibit marked fibrotic parenchymal alterations, although with less areas of fibrotic lesions than observed in the vehicle treated bleomycin-induced fibrotic lungs.

## Discussion

We demonstrate XLF as a novel and sophisticated technique to preclinically assess lung function non-invasively overtime that can be performed in combination with microCT and WBP to monitor the severity of bleomycin induced pulmonary fibrosis as well as the efficacy of the treatment response to the tyrosine kinase inhibitor Nintedanib. Lung dysfunction following bleomycin instillation was successfully reflected in the XLF in-vivo functional measurements performed in this study by a significant reduction in the decay rate of the expiration phase. This also indicates, that the functional abnormalities in the initial inflammatory and fibrotic phase, occur as early as day 7, which continue to decline in the absence of therapeutic intervention. Our data for the XLF measurements showed a decline in the decay rate from day 7 until day 14 in the vehicle treated mice with a detected improvement on day 21. The parameter decay rate is predominantly affected by alterations in the lung compliance and the observed increase in decay rate suggests that by day 21 the vehicle treated mice with severe LF might show the onset of the self-healing mechanism as for instance described by Tashiro et al.^19^. However, this self-healing process is far from being complete as evidenced by the presence of fibrotic regions in the subsequently performed histological analysis. This suggests that lung function could already be improved at day 21 despite the presence of fibrotic alterations in the lung. We postulate that these improved lung function findings are partially due to the recruitment of the intercostal muscles as a sign of forced expiration which could work as a compensatory mechanism. Further evidence can be found in supplemental material S3. This notion is further supported by our WBP data which also presented an increase in the tidal volume on day 21. The found improvement of the lung function in the untreated group might be also related to the fact that we used young mice of approx. thirteen weeks of age. Jenkins et al.^20^ showed that using aged 18-24 months old mice could prevent spontaneous resolution or regeneration after lung injury and mimics human-like pulmonary fibrosis pathophysiology. Nevertheless, a relentlessly disrupted breathing cycle in the vehicle treated LF mice is clearly evident in our XLF acquired x-ray transmission curve which showed a low peak inhalation and high expiration baseline on day 21. This further suggests that fibrosis induced stiffening of lung parenchyma in the untreated LF mice increases elastance and reduces lung compliance causing shortness of breath overtime as described by Devos et al.^21^. Moreover, Devos et al.^21^ report in the same publication force expiration measurements which are well in line with our findings. A previous terminal in vivo study by Manali et al. performed on a bleomycin induced C57BL/6 mouse model of LF also showed a significant increase in pressure-volume area (PV-A) which represents lung elastics hysteresis as early as one week after bleomycin instillation which worsened by day 21^12^. Importantly, we performed XLF measurements at multiple time points on the same animal while Manali et al. used forced oscillation technique which required sacrificing the animals after lung function measurement due to tracheotomy and muscle paralysis at each time point, preventing longitudinal assessment and substantially increasing the number of animals required^12^.

The attenuation of fibrosis progression due to therapeutic intervention is clearly evident from the decay rate in the Nintedanib treated LF group. In comparison to the vehicle treated mice with LF, all parameters including decay rate, $V^{insp}_{\mu CT}$ and $TV_{WBP}$ showed an enhanced functional capacity in the Nintedanib treated LF group one week after treatment. This Nintedanib induced functional recuperation can be attributed to its anti-fibrotic and anti-inflammatory activity which has been previously reported in animal models^2^. For instance, the anti-fibrotic role of Nintedanib treatment was previously demonstrated by reduced tissue density in microCT, histological score, mRNA expression of TGF-$\beta$1 and pro-collagen 1 as well as improved lung function and compliance in comparison to controls^2,14,22^. Additionally, the anti-inflammatory activity was previously presented by an overall decreased accumulation of lymphocytes and neutrophils in bronchoalveolar lavage fluid^3^. Both of these modes of action might have contributed to an enhanced decay rate and lung volume capacity in LF mice that received Nintedanib treatment as shown in our study. Nonetheless, Nintedanib failed to completely restore full functional capability of lungs as reflected by the lower peak of inhalation in the Nintedanib treated LF mice than in the healthy group on day 21. The expiratory baseline, however, was restored almost completely and similar to that of the healthy group which is in agreement with a previous study that reported a significant improvement in forced expiration following Nintedanib treatment in a bleomycin induced mouse model of LF^23^.

High resolution CT (HRCT) is the clinical standard for diagnostics of lung fibrosis^24^. We performed microCT as a gold standard technique for the in-vivo assessment of lung gas volume which showed significantly lower air-filled spaces in vehicle treated LF mice than the Nintedanib treated LF and healthy mice. The anti-fibrotic effect of Nintedanib to significantly inhibit the increase in poorly aerated tissue has been previously shown using microCT^14,25^. Notably, XLF relies on the fast acquisition of low dose radiographic measurements, thus making it more suitable than microCT for longitudinal assessment. WBP also presented an enhanced lung function in Nintedanib treated mice in comparison to the vehicle treated group. As compared to XLF, WBP however, is complicated to perform requiring chamber habituation, standardization of room lighting and time of the day for reproducible results^26^.

In contrast to Pannati et al.^25^ in which ventilation defects were extracted from post-processed retrospectively gated microCT data sets only, we show a strong correlation between in-vivo volumetric parameters including $V^{insp}_{\mu CT}$ and $TV_{WBP}$ and also with ex-vivo high-resolution SR$\upmu$CT for all groups. This reflects the high reliability of our obtained XLF data. In mice, dense accumulation of tissue components including collagen is one of the hallmarks of lung fibrosis^27^ which was also observed in the ex-vivo SR$\upmu$CT and histology data presented in this study for the vehicle treated group. Moreover, Nintedanib induced retardation of fibrotic progression was also evident in the ex-vivo data which showed higher mean air ratio in SR$\upmu$CT data and lower histological score than the vehicle treated mice. Although the lung function was improved, the histology still showed unresolved fibrotic areas in the Nintendanib treated LF mice, suggesting that while the lung function had markedly improved, the fibrosis was not completely resolved by day 21.

In conclusion, our study highlights that XLF measurement is a simple and reliable technique to not only assess fibrosis progression but also for monitoring the treatment response in the bleomycin mouse model of LF. We suggest that XLF could be used as a more user-friendly and faster alternative to WBP for assessing lung function in various pharmacological efficacy mouse studies. Importantly, XLF by using a lower x-ray dose and faster acquisition time than CT, is more suitable for longitudinal pre-clinical application. Lastly, with the emergence of new pulmonary diseases such as Covid-19^28^ and Middle Eastern Respiratory Syndrome (MERS)^29^, XLF as a novel preclinical technique can efficiently provide functional readouts over the course of the disease in animal models.

## Methods

### Animal experiment

Thirteen weeks old male C57Bl/6 mice, with an average weight of 26.3 ± 1.5 g, were used for induction of fibrosis and x-ray based in-vivo lung function and CT measurements (Table S1). The mice were housed in a temperature and humidity-controlled room with a 12 h light-dark cycle throughout the experiments and were fed standard rodent chow and water ad libitum. Animals were randomly divided into three groups: healthy (N=4), bleomycin (bleo.) + vehicle treated (N = 5) and bleo. + Nintedanib treated group (N = 7). On day 0, baseline XLF, WBP and microCT measurements were performed in all groups. On the same day, mice belonging to the vehicle and Nintedanib treated lung fibrosis groups were anesthetized and intra-tracheally (i.t.) administered with a single dose of 50 $\upmu$l of bleomycin (1.5 mg/kg, BLEO-cell, STADA Pharma, GmbH). The healthy group did not receive any i.t. treatment. One week after induction of fibrosis at day 7, following XLF and microCT measurements, 60 mg/kg of Nintedanib dissolved in 200 $\upmu$l 1 % Tween 80-PBS was administered to the treated group via oral gavage daily until day 21, based on Ruscitti et al.^14^. The vehicle treated group received 200$\upmu$l of 1% Tween 80-PBS. In-vivo XLF, WBP and microCT were performed in all groups on day 14 and day 21. After the correlative lung function measurements, mice were sacrificed by cervical dislocation on day 21 (Fig. 1). Lungs were excised for further assessment by high-resolution synchrotron radiation (SR) based microCT and histology.

### In-vivo XLF measurement

XLF was performed on mice anesthetized using approximately  2 % isoflurane in 1 L 50/50 mix of oxygen and air per min. Radiographic x-ray images of the mouse chest cavity were acquired using a low-dose in-vivo microCT (QuantumFX, PerkinElmer) as previously described by Dullin et al.^15^ and Khan et al.^18^. Briefly, the mice were placed inside a custom-made plethysmography chamber and the breathing rate of the mice was manually adjusted by altering the isoflurane concentration between 1.5 and 3% to achieve and maintain a breathing frequency of 0.7 Hz. Radiographic images for XLF were acquired using an x-ray tube voltage of 90 kVp and a tube current of 100 $\upmu$A resulting in an average x-ray dose of approximately 37 mGy. The images of the chest motion obtained during the active breathing were sequenced to produce a movie and the lung region was used for evaluating various parameters of XLF, mainly the decay rate in the expiration phase.

### In-vivo microCT imaging

MicroCT (QuantumFX, PerkinElmer) was performed on the isoflurane-anesthetized mice using the following parameters: x-ray tube voltage = 90 kVp, tube current = 200$\upmu$A. Radiographic images were acquired for 360$^\circ$ gantry rotation for a total scan time of 270 s (resulting in an x-ray dose of approximately 290 mGy). The entire microCT scan was retrospectively gated^30^.

### In-vivo microCT data processing and lung volume evaluation

MicroCT reconstruction was performed using retrospective gating and a modified Feldkamp (FDK) reconstruction algorithm as previously described in Khan et al.^18^. Briefly, at a position close to the diaphragm the average x-ray attenuation was measured on one line and at all time points. This function shows temporal variations related to the breathing motion of the mouse. A moving average filtered curve was subtracted to correct the baseline of the signal. From the resulting breathing curve, a number of frames detected per inhaled and exhaled phase were selected to generate two segregated sets of projections for each breathing phase, which were then reconstructed individually. To further enhance the quality of the resulting 3D data sets for each breathing phase, a slice below the lung region was subtracted from all slices to produce a uniform background. In addition, a 3D Gaussian-filter was applied to reduce the noise level. The lung region from the filtered 3D volumes at each inspiration and exhalation phase was masked to exclude the surrounding thoracic cavity. Segmentation of air-filled lung regions from dense tissue structures was performed using a region growing method. A closing operator was applied on the resulting lung mask to close the gaps mostly related to the presence of bigger vessels. The lung volume $V_{\mu CT}$ for both inspiration $V^{insp}_{\mu CT}$ and expiration $V^{exp}_{\mu CT}$ phase was estimated by multiplying the number of segmented voxels with the volume per voxel resulted in the lung volume.

### Whole-body plethysmography

WBP was performed using a customized chamber that fitted inside the gantry of the microCT^18^. We recorded the pressure differences between the recording chamber (220 ml) and a reference chamber (50 ml) via a differential pressure sensor (DPS, Board Mount Pressure Sensor, 0–1” H_2_O, 20 mV, 16 VDC supply; Mfr. No: INCH-D-MV, Amphenol Cooperation Wallingford, CT, USA). Analog-digital interface (PowerLab) and LabChart-software were used for digitalization of the data obtained. The raw flow signal was bandpass filtered (0.5–20 Hz) to remove motion artifacts, noise as well as bias flow, and the resulting signal was then integrated for measuring a tidal volume ($TV_{WBP}$). We used the *Integral Channel Calculation module* of the LabChart-software in which we included all data points, reset each cycle, wherein the integral is reset every time the source signal passes through zero to a positive value. We used LOG32 data logger (Dostmann electronic GmbH, Wertheim-Reicholzheim, Germany) to measure the temperature and humidity inside the plethysmography chamber, according to the protocol described in Khan et al.^18^.

### Ex-vivo imaging by phase-contrast synchrotron radiation $\upmu$CT (SR$\upmu$CT)

Following the last lung function measurements, mice were euthanized and the lungs were excised. The explanted lungs were inflated with a cannula through the trachea by gentle infusion with 1 ml of 4% paraformaldehyde (PFA) and subsequently fixed for 24 h. The fixed lungs were chemically dehydrated using an ascending ethanol series and embedded in paraffin. The paraffin embedded lungs were imaged at the SYRMEP beamline (Synchrotron Light Source Elettra, Trieste, Italy) using a white beam setup^31^ with following parameters: average photon energy E = 23.6 keV, 360$^\circ$ off-center scan with 1800 angular projections, detector exposure time per projection 20 ms, and a sample-to-detector distance of 150 mm to manifest the phase effects. A single-distance phase-retrieval algorithm was applied with a $\delta -to-\beta$ ratio of 100, to generate 3D data sets with a voxel size of 2 $\times$ 2 $\times$ 2 $\upmu$m$^{3}$ which predominately expressed the distribution of the phase shift component “$\delta$” of the complex refractive index within the lung. This was done to enhance the contrast of the soft-tissue i.e., the lung as previously shown by Saccomano et al.^32^.

### Histological staining

Following SR$\upmu$CT imaging, the paraffin-embedded lungs were cut into series of 2 $\upmu$m thick sections. 10–12 sections per lung from representative regions were deparaffinized and stained as described before with hematoxylin and eosin (H &E) and Masson trichrome staining (MTS) for assessment of collagen^33,34^. The Ashcroft score^35^ for the fibrotic burden ranging from 0 to 8 was used for precise determination of the extent of fibrosis, ranging from 0 no collagen to 8 high amount of collagen. The scoring was performed in a blind and randomized manner by three independent readers. The final score for each mouse was calculated as median between the observers and as average of all histological slices analyzed per mouse.

## Ethical statement

All animal procedures were performed in compliance with the guidelines of the German ethical laws, were approved by the administration of Lower Saxony, Germany (approval number G17.2585) and are reported in accordance to the ARRIVE guidelines (https://arriveguidelines.org).

### Data quantification and statistical analysis

Lung volumes from microCT data were quantified using RetrospeCT https://gitlab.gwdg.de/thomas.rittmann/retrospective_gating. Quantification of XLF measurements were achieved with a customized software xLFinal https://github.com/xPITcoding/xLFinal.git. Digitalization and analysis of WBP data was performed with an analog-digital interface (PowerLab) and LabChart-software (ADInstruments). SR$\upmu$CT data was reconstructed using SYRMEP Tomo Project (STP) https://github.com/ElettraSciComp/STP-Gui and processed using VGSTUDIO MAX (Volume Graphics, GmbH). For the quantification of the lung morphology at selected regions of interest (ROIs), surface determination was performed in VGSTUDIO MAX software to calculate the air and tissue volume and surface area. For statistical analysis, the multiple comparison unpaired t-test implemented in GraphPad Prism Software version 9 was used (GraphPad Software, La Jolla California USA) with a *p* of 0.05 (*) as margin for statistical significance. Correlation coefficient was determined by using Pearson correlation coefficient and Spearman rank correlation coefficient (GraphPad Software, version 9).

### Supplementary Information

Supplementary Information.

## Data Availability

Due to the large size of especially the synchrotron data, the data cannot be hosted at an online repository. Thus, the data is available under reasonable request from the authors (christian.dullin@med.uni-goettingen.de).

## Abbreviations

WBP: Wholebody plethysmography
CT: Computed tomography
LF: Lung fibrosis
XLF: X-ray based lung function measurement
SR$\upmu$CT: Synchrotron based micro computed tomography
HRCT: High resolution computed tomography
LF: Lung fibrosis
bleo.: Bleomycin
AUC: Area under the curve
H&E: Haematoxylin eosin staining
MTS: Masson Goldner’s trichrome staining
3D: Three dimensional

## References

[1] Jenkins, G. *Demystifying Pulmonary Fibrosis* (Springer, 2020). 10.1152/ajplung.00365.2020 PMC7839634 32755321

[2] Wollin L (2015). Mode of action of nintedanib in the treatment of idiopathic pulmonary fibrosis. Eur. Respir. J..

[3] Wollin L, Maillet I, Quesniaux V, Holweg A, Ryffel B (2014). Antifibrotic and anti-inflammatory activity of the tyrosine kinase inhibitor nintedanib in experimental models of lung fibrosis. J. Pharmacol. Exp. Therapeut..

[4] Umemura Y (2021). Efficacy and safety of nintedanib for pulmonary fibrosis in severe pneumonia induced by covid-19: An interventional study. Int. J. Infect. Dis..

[5] Carrington R, Jordan S, Pitchford S, Page C (2018). Use of animal models in ipf research. Pulmon. Pharmacol. Therapeut..

[6] Nygaard K, Smith-Erichsen N, Hatlevoll R, Refsum SB (1978). Pulmonary complications after bleomycin, irradiation and surgery for esophageal cancer. Cancer.

[7] Liu Y, Keikhosravi A, Mehta G, Drifka C, Eliceiri K (2017). Fibrosis: Methods and protocols. Methods Mol. Biol..

[8] Della-Latta V, Cecchettini A, Del-Ry S, Morales M (2015). Bleomycin in the setting of lung fibrosis induction from biological mechanisms to counteractions. Pharmacol. Res..

[9] Adler A, Cieslewicz G, Irvin CG (2004). Unrestrained plethysmography is an unreliable measure of airway responsiveness in balb/c and c57bl/6 mice. J. Appl. Physiol..

[10] Lundblad LK, Irvin CG, Adler A, Bates JH (2002). A reevaluation of the validity of unrestrained plethysmography in mice. J. Appl. Physiol..

[11] Hülsmann S (2021). Evaluation of a mechanical lung model to test small animal whole body plethysmography. Sci. Rep..

[12] Manali ED (2011). Static and dynamic mechanics of the murine lung after intratracheal bleomycin. BMC Pulmon. Med..

[13] Ruscitti F (2017). Longitudinal assessment of bleomycin-induced lung fibrosis by micro-ct correlates with histological evaluation in mice. Multidiscipl. Respir. Med..

[14] Ruscitti F (2020). Quantification of lung fibrosis in ipf-like mouse model and pharmacological response to treatment by micro-computed tomography. Front. Pharmacol..

[15] Dullin C (2016). X-ray based lung function measurement-a sensitive technique to quantify lung function in allergic airway inflammation mouse models. Sci. Rep..

[16] Markus MA (2017). X-ray-based lung function measurement reveals persistent loss of lung tissue elasticity in mice recovered from allergic airway inflammation. Am. J. Physiol.-Lung Cell. Mol. Physiol..

[17] Dullin C, Svetlove A, Zschüntzsch J, Alves F (2022). Simultaneous assessment of lung morphology and respiratory motion in retrospectively gated in-vivo microct of free breathing anesthetized mice. Sci. Rep..

[18] Khan A (2021). Simple low dose radiography allows precise lung volume assessment in mice. Sci. Rep..

[19] Tashiro J (2017). Exploring animal models that resemble idiopathic pulmonary fibrosis. Front. Med..

[20] Jenkins RG (2017). An official american thoracic society workshop report: Use of animal models for the preclinical assessment of potential therapies for pulmonary fibrosis. Am. J. Respir. Cell Mol. Biol..

[21] Devos FC (2017). Forced expiration measurements in mouse models of obstructive and restrictive lung diseases. Respir. Res..

[22] Martinez FJ (2017). Idiopathic pulmonary fibrosis. Nat. Rev. Dis. Prim..

[23] Anzulovic, Z. *et al*. A translational value of pulmonary function tests in a mouse model of bleomycin-induced pulmonary fibrosis: Effects of approved therapies Nintedanib and Pirfenidone. In *ILD/DPLD of known origin* PA4727. 10.1183/13993003.congress-2019.PA4727 (European Respiratory Society, 2019).

[24] Christe A (2019). Computer-aided diagnosis of pulmonary fibrosis using deep learning and ct images. Investig. Radiol..

[25] Pennati F (2023). Micro-ct-derived ventilation biomarkers for the longitudinal assessment of pathology and response to therapy in a mouse model of lung fibrosis. Sci. Rep..

[26] Quindry JC, Ballmann CG, Epstein EE, Selsby JT (2016). Plethysmography measurements of respiratory function in conscious unrestrained mice. J. Physiol. Sci..

[27] Walters DM, Kleeberger SR (2008). Mouse models of bleomycin-induced pulmonary fibrosis. Curr. Protocols Pharmacol..

[28] Velavan TP, Meyer CG (2020). The covid-19 epidemic. Trop. Med. Int. Health.

[29] Azhar EI, Hui DS, Memish ZA, Drosten C, Zumla A (2019). The middle east respiratory syndrome (mers). Infect. Dis. Clin..

[30] Bartling SH (2007). Retrospective motion gating in small animal ct of mice and rats. Investig. Radiol..

[31] Dullin C (2021). Multiscale biomedical imaging at the syrmep beamline of elettra-closing the gap between preclinical research and patient applications. Phys. Open.

[32] Saccomano M (2018). Synchrotron inline phase contrast $$\mu$$ct enables detailed virtual histology of embedded soft-tissue samples with and without staining. J. Synchr. Radiat..

[33] Foot NC (1933). The masson trichrome staining methods in routine laboratory use. Stain Technol..

[34] Cardiff RD, Miller CH, Munn RJ (2014). Manual hematoxylin and eosin staining of mouse tissue sections. Cold Spring Harbor Protoc..

[35] Ashcroft T, Simpson JM, Timbrell V (1988). Simple method of estimating severity of pulmonary fibrosis on a numerical scale. J. Clin. Pathol..

