# Supplementary material for: Longitudinal x-ray based lung function measurement for monitoring Nintedanib treatment response in a mouse model of lung fibrosis
Source: Sci Rep. 2023 Oct 30;13:18637. doi: 10.1038/s41598-023-45305-x (PMC10616088; doi:10.1038/s41598-023-45305-x)
Supplement: Supplementary file 1 — Supplementary Information. [file 41598_2023_45305_MOESM1_ESM.docx]

**Monitoring the response to Nintedanib treatment in a mouse model of lung fibrosis using longitudinal X-ray based lung function measurement**

Amara Khan^1^, Andrea M. Markus^1^, Angelika Svetlove^1,2^, Swen Hülsmann^3^, Frauke Alves^1,2,4,5^ and Christian Dullin^1,5,6,7*^

**S1**: **Animal data:**

The mice used for correlative lung function and volume measurements were all age (13 weeks), strain (C57Bl/6) and gender (male) matched. This was done to reduce the number of factors that may influence the lung function measurements. All mice were assigned.

**Table S1: Mouse body weights and treatment groups.**

| Mouse no. | Body weight (g)  on baseline (Day 0) | Group |
| --- | --- | --- |
| 1 | 26.6 | Healthy |
| 2 | 25.0 | Healthy |
| 3 | 24.9 | Healthy |
| 4 | 28.2 | Healthy |
| 5 | 26.0 | Treated |
| 6 | 25.6 | Treated |
| 7 | 25.0 | Treated |
| 8 | 27.6 | Treated |
| 9 | 24.0 | Treated |
| 10 | 25.6 | Treated |
| 11 | 26.2 | Treated |
| 12 | 29.0 | Untreated |
| 13 | 26.0 | Untreated |
| 14 | 25.2 | Untreated |
| 15 | 27.7 | Untreated |
| 16 | 28.4 | Untreated |

**S2: Overview histological appearance of the different mice**


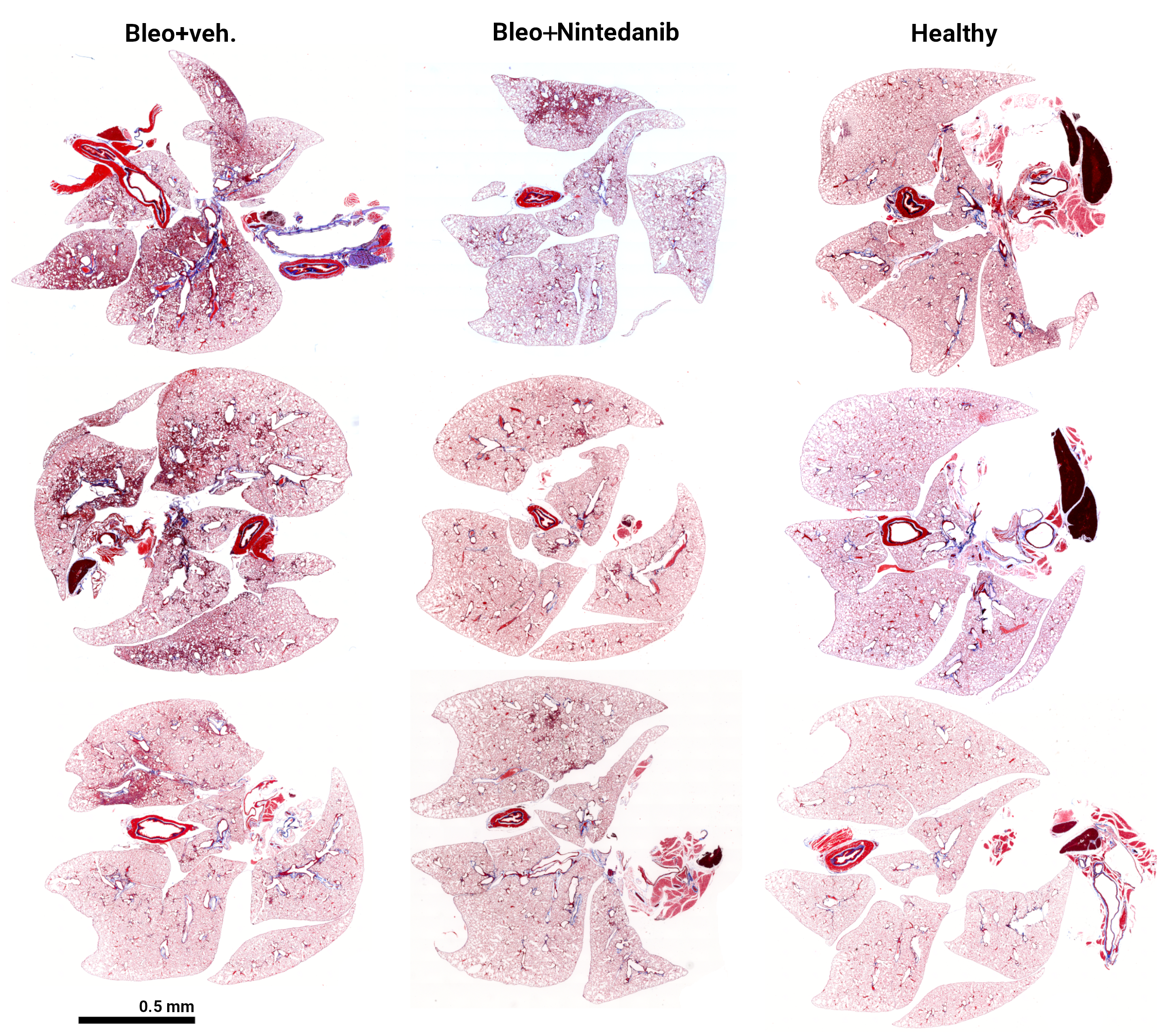
Examplarly MTS stained lung slices of three mice per group are show to demonstrate the heterogeneity of the fibrosis model as well as the differences in the treatment response. The left column (Bleo+veh.) shows three untreated mice. A patchy distribution of fibrotic regions can be seen. In addition, the third example clearly shows a lower severity of fibrosis. However, the Nintedanib treated mice (middle column) overall show a lower amount of fibrotic regions. In all the healthy controls (right column) no obvious fibrotic regions can be found. All mice were sacrificed at the same time point: 21 days after bleomycin induction and 14 days after Nintedanib application. All slices are shown in the same magnification (bar represents 0.5mm)

**S3: Recruitement of intercostal muscle in fibrotic mice during the breathing process**


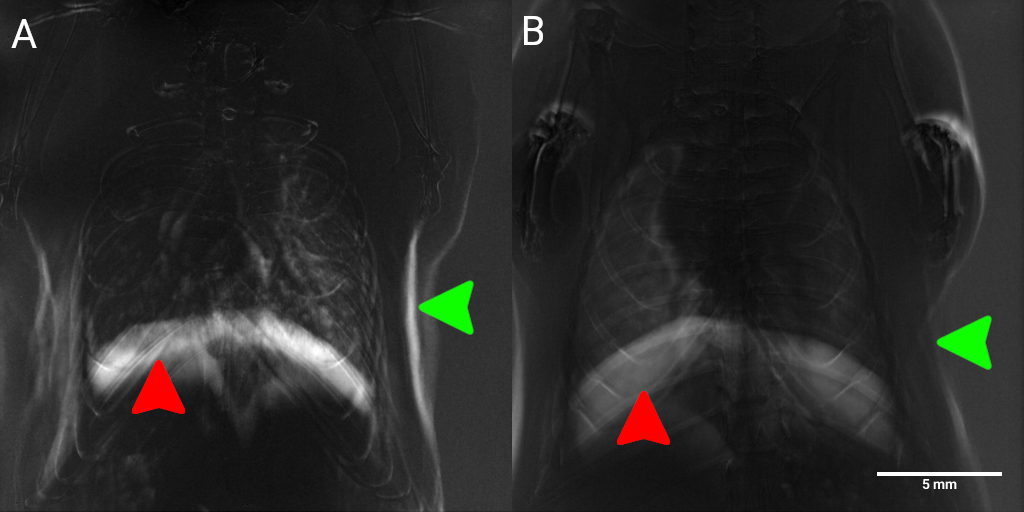
Pixelwise Standarddeviation over time in cinematic planar x-ray imaging. Bright areas represents regions with strong movement over time. A) shows a fibrotic mouse while B) shows an healthy mouse. Clearly the motion of the diaphragm (red arrowhead) is reduced in the fibrotic animal compared to the healthy control. In addition, the fibrotic animal shows an increased motion of the costal muscle regions of the chest (green arrowhead) suggesting forced expiration. (Roughly 20 breathing events with a breathing frequency of approximately 0.7 Hz were averaged to generate this data)
